# Acupuncture against chronic postsurgical pain in non-small cell lung cancer patients: A protocol of randomized controlled trial

**DOI:** 10.1097/MD.0000000000027461

**Published:** 2021-10-08

**Authors:** Gang Li, Changxi Zhang, Congyi Wang, Ling Xiao

**Affiliations:** Department of Thoracic Surgery, Chongqing Steel General Hospital, Dadukou District, Chongqing, China.

**Keywords:** acupuncture, lung cancer, pain, video-assisted thoracoscopic lobectomy

## Abstract

**Introduction:**

Video-assisted thoracoscopic lobectomy is the prior recommended treatment for non-small cell lung cancer (NSCLC), with the advantages of small trauma, less postoperative pain, and quick recovery. However, a large number of patients may suffer chronic postsurgical pain (CPSP), which makes the patients unwilling to practice pulmonary exercises, and it would directly affect patient's cough, sputum expectoration, and mobility. Opioids could greatly improve the quality of postoperative analgesia and the quality of life after surgery, but it is accompanied with obvious side effects. A number of clinical studies have proved that acupuncture could improve postoperative pain and reduce opioid use. In this study, we try to conduct a randomized controlled study to evaluate the efficacy and safety of plum-blossom needle acupuncture combined with Tramadol in improving CPSP after lobectomy in NSCLC patients.

**Methods:**

Patients will be randomly divided into treatment group (acupuncture plus Tramadol) and control group (sham acupuncture plus Tramadol) with a random number table in 1:1 ratio. The patients, outcome assessor, and statistician will be blinded. The outcomes are changes of numerical rating scale, Karnofsky performance score, brief pain inventory, blood routine, liver and kidney function. The data will be analyzed by SPSS 22.0.

**Conclusions:**

The results will help to evaluate the efficacy and safety of plum-blossom needle acupuncture in improving CPSP after lobectomy in NSCLC patients.

## 1 Introduction

Lung cancer is one of the most common malignant tumors, with the highest morbidity and mortality rate worldwide, which seriously threatens human health and life.^[1–3]^ Non-small cell lung cancer (NSCLC) is the major type, and lobectomy is one the effective treatments for early NSCLC.^[4]^ Nowadays, the rapidly emerging technique, video-assisted thoracoscopic lobectomy (VATL) is the prior recommended treatment for NSCLC, with the advantages of small trauma, less postoperative pain, quick recovery, and better patient satisfaction.^[5–8]^ Recently, VATL continues to improve with the decreasing working port size and incisions. The conventional multiportal VATL with several incisions was alternated to triportal, and recently biportal, or even uniportal VATL.^[9,10]^

However, in patients undergoing lobectomy, a large number would suffer chronic postsurgical pain (CPSP), the most common complication after chest surgery. The biportal or uniportal VATL approach has been proposed to perform feasibly and safely in lobectomy with less incisions and quicker recovery, but many patients may still complicate with CPSP.^[11–13]^ The occurrence and severity of CPSP after lobectomy is an important factor to influence the recovery of patients with NSCLC. CPSP makes the patients unwilling to practice pulmonary exercises, and it directly affects patient's cough, sputum expectoration, and mobility. Patient with CPSP may even complicate with atelectasis, lung infection, hypoxemia, which greatly lower the quality of life.^[12,14]^

Conventional analgesia methods for CPSP after lobectomy mainly include patient controlled analgesia, regional analgesia, and analgesic pumps. Patient controlled analgesia, involving opioids such as morphine and fentanyl, as well as anti-inflammatory drugs, could greatly improve the quality of postoperative analgesia and the quality of life after surgery, but it is accompanied with obvious side effects, such as urine retention, vomiting, and allergies. Therefore, to seek a safe and effective combined therapy is important for the treatment of CPSP.

Acupuncture, a traditional alternative therapy, has been used to treat pain for many years,^[15]^ and a number of clinical studies have proved its efficacy for postsurgical pain. Acupuncture could improve postoperative pain and reduce opioid use.^[16]^ However, there are fewer studies on acupuncture against CPSP after lobectomy. Our group preliminarily found that plum-blossom needle percussion, one of the acupuncture therapies in traditional Chinese medicine, combined with analgesic could significantly relieve CPSP in NSCLC patients and reduce the side effects of analgesic. Therefore, in this study, we try to conduct a prospective, randomized controlled study to evaluate the efficacy and safety of plum-blossom needle combined with Tramadol Hydrochloride Sustained Release Tablets in improving CPSP after lobectomy in NSCLC patients.

## 2 Methods

### 2.1 Patients selection

The randomized, controlled trial will be carried out in Chongqing Steel General Hospital, and it has been approved by the Health Research Ethics Board of Chongqing Steel General Hospital. The study has been registered in open science framework with registration number of DOI 10.17605/OSF.IO/RJEYB. All patients will provide a signed written informed consent at enrollment.

The inclusion criteria for participants are as follows:

(1) diagnosis of CPSP after lobectomy in NSCLC patients;
(2) being aged between 35 and 75 years old;
(3) with 3 < numerical rating scale (NRS) < 7;
(4) with Karnofsky performance score > 60;
(5) with signed written informed consent.

Patients will be excluded with the following criteria:

(1) with previous history of thoracic surgery;
(2) who had undergone targeted therapy or chemoradiotherapy before;
(3) with peripheral structures invasion;
(4) with contraindication plum-blossom needles, such as wounds, ulcers, scars at the puncture site;
(5) with abnormal renal or liver function;
(6) with other surgical contradictions.

### 2.2 Randomization and blinding

**Figure 1:**
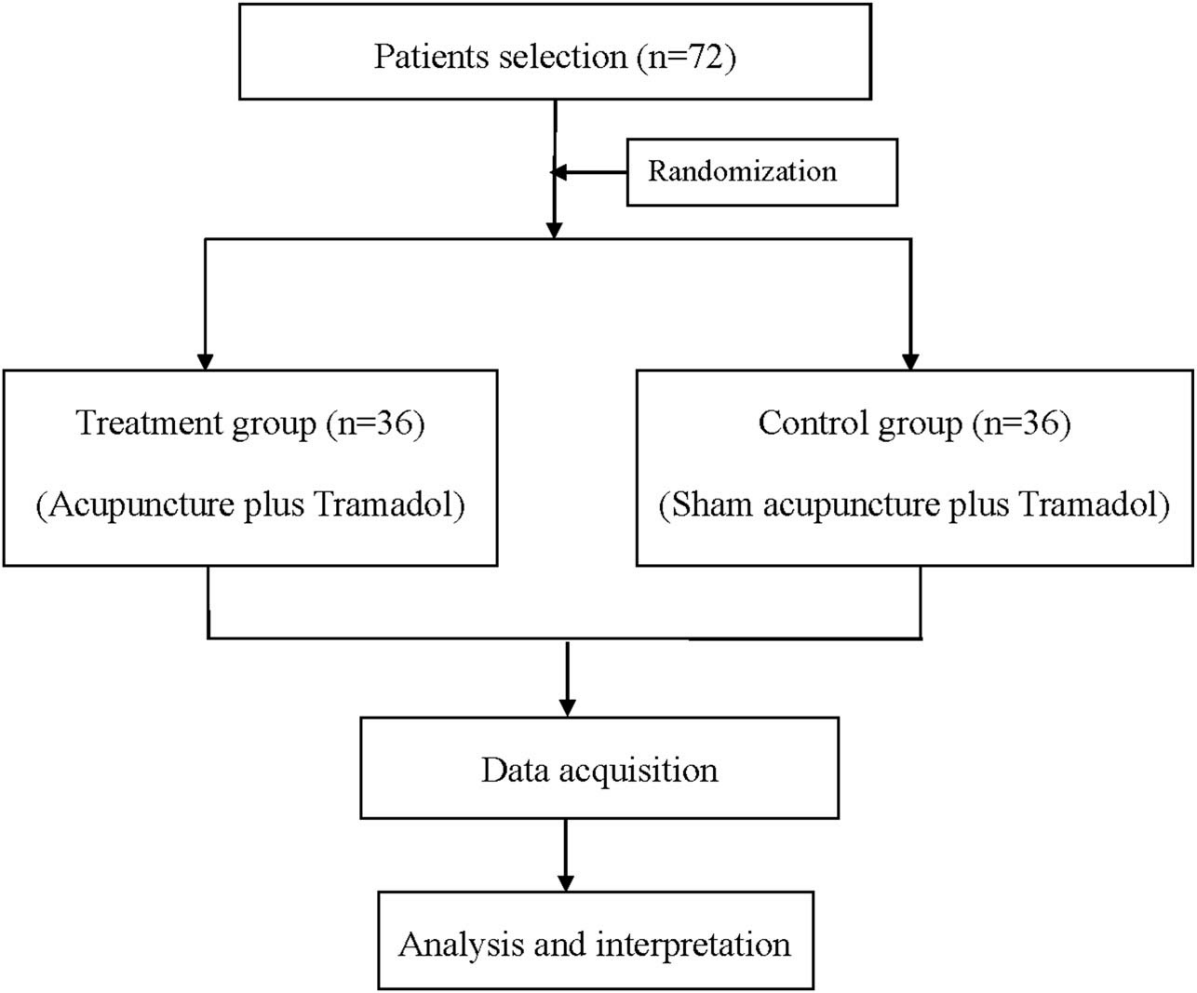
Flow diagram.

Patients will be randomly divided into treatment group (acupuncture plus Tramadol) and control group (sham acupuncture plus Tramadol) with a random number table in 1:1 ratio (Fig. 1). To maintain blinding, patients in both groups will be performed plum-blossom needles percussion. In the treatment group, the plum-blossom needles will be stick on the pain area, while in the control group, the needles will be performed around the pain area as sham acupuncture. The patients, outcome assessor, and statistician will be blinded.

### 2.3 Interventions

Patients with CPSP in both groups will take Tramadol Hydrochloride Sustained Release Tablets (Shandong Xinhua Pharmaceutical Company Limited, H19990062), 100 mg, and twice a day for 14 days.

In the treatment group, plum blossom needles (Gushi Medical Instrument Co., LTD, 20192200580) percussion will be performed on pain area, 70 to 90 times per minute for 10 minutes, with moderate percussion intensity, once every 2 days, and the treatment course will be 14 days.

While in the control group, plum blossom needles (Gushi Medical Instrument Co., LTD, 20192200580) percussion will be performed around the pain area, 70 to 90 times per minute for 10 minutes, with moderate percussion intensity, once every 2 days, and the treatment course will be 14 days.

### 2.4 Outcome measures

The primary outcome is the change of NRS at day 0, day 7, and day 14. The secondary outcomes are Karnofsky performance score, brief pain inventory, blood routine, liver and kidney function before and after the treatment. The change of Tramadol application will be recorded. All adverse events will be also recorded.

### 2.5 Sample size

In our preliminary trial, the changes of NRS in treatment group and control group are 2.65 ± 1.25 and 1.8 ± 1.31, respectively. Taking α = 0.05, β = 0.2, 30 cases will be needed in each group. Considering a loss to follow-up of 20%, the estimated sample size will be 72.

### 2.6 Statistical methods

The data will be analyzed by SPSS 22.0 (IBM, Chicago). The count data will be described by number and percentages, and the comparison between groups will be performed by chi-square test; The measurement data will be presented as mean ± standard deviation. Repeated measures analysis of variance will be applied for repeated data, considering normality and homogeneity. For the changes between groups, *t* test or Mann–Whitney *U* test will be applied accordingly. *P* < .05 will be considered statistically significant.

## 3 Discussions

Lobectomy is an important treatment for patients with NSCLC lung cancer. With the increasing enhancement of equipment and technology, VATL could greatly lower the incidence of mortality and complications.^[7,17,18]^ However, patients may still suffer from CPSP. CPSP refers to the continuous or intermittent pain, mainly caused by surgery. In most cases, CPSP lasts longer than 2 months, and it could seriously affect the rehabilitation and quality of life of patients.^[19]^ CPSP could lower the respiratory rate and tidal volume to cause atelectasis and pulmonary infection. Opioids, such as Tramadol, could improve the quality of postoperative analgesia and the quality of life after surgery, but it is accompanied with obvious side effects.

Many studies have showed that acupuncture can be useful for the postoperative pain management^[20]^ and reduced opioid use.^[16]^ Plum blossom needle is an important acupuncture method in traditional Chinese medicine, and it has the advantages of low cost, high tolerance, convenience, and minimal adverse reactions.^[21]^ In this study, we try to conduct a prospective, randomized controlled study to evaluate the efficacy and safety of plum-blossom needle combined with Tramadol Hydrochloride Sustained Release Tablets in improving CPSP after lobectomy in NSCLC patients. Also, there are several potential limitations of the trial. First, the single-blinded design may cause inevitable bias. Second, the limited patients’ source may cause some biases.

## Author contributions

**Data collection:** Congyi Wang and Ling Xiao.

**Data curation:** Congyi Wang.

**Funding acquisition:** Gang Li.

**Funding support:** Gang Li.

**Investigation:** Congyi Wang and Ling Xiao.

**Literature retrieval:** Changxi Zhang and Congyi Wang.

**Software operating:** Congyi Wang.

**Supervision:** Ling Xiao.

**Writing – original draft:** Gang Li and Changxi Zhang.

**Writing – review & editing:** Gang Li and Gang Li.
